# Predictors and Early Outcomes of Hidden Blood Loss Following Surgery for Spinal Metastases: A Retrospective Study Focusing on Tomita Type 1–5 Lesions

**DOI:** 10.3390/jcm15041356

**Published:** 2026-02-09

**Authors:** Xinyao Lv, Ruizhao Zhao, Yuyu Fan, Zijian Wang, Junjie Qiao, Xiutong Fang

**Affiliations:** Department of Spinal Surgery, Beijing Shijitan Hospital, Capital Medical University, Beijing 100038, China; 15129528233@163.com (X.L.); ruizhao11@163.com (R.Z.); fanyy0507@163.com (Y.F.); 15652283881@163.com (Z.W.)

**Keywords:** hidden blood loss, risk factors, spinal metastasis surgery, multiple regression analysis, enhanced recovery after surgery

## Abstract

**Background**: Hidden blood loss (HBL) following surgery for spinal metastases constitutes a major portion of total blood loss (TBL), yet its predictors and impact on early recovery remain unclear. This study aimed to identify independent predictors of HBL in patients with Tomita type 1–5 lesions and to assess its association with early clinical outcomes. **Methods**: In this retrospective study of 230 patients undergoing posterior tumor resection with cement augmentation and fixation, HBL was calculated using the Gross equation. Predictors were identified via univariate and multivariate linear regression. The impact of HBL on postoperative length of stay, change in Karnofsky Performance Status (ΔKPS), moderate-to-severe anemia, and complications was evaluated using adjusted regression models. Additionally, receiver operating characteristic curve analysis was performed to explore the predictive value of HBL for adverse events. **Results**: Mean HBL was 449.87 ± 284.86 mL (37.1% of total loss). Independent predictors included higher body mass index (BMI), longer surgery, extensive vertebral involvement (Tomita 4–5), and preoperative hypertension (all *p* < 0.05). Higher HBL independently predicted longer hospital stay (β = 0.023, *p* < 0.001), worse ΔKPS (β = −0.012, *p* < 0.001), increased anemia risk (OR = 1.002, *p* < 0.001), and more complications (OR = 1.003, *p* < 0.001). Receiver operating characteristic curve analysis suggested that a HBL >382.5 mL was associated with an increased risk of complications requiring intervention, and a HBL >344.0 mL was associated with an increased risk of postoperative moderate-to-severe anemia. **Conclusions**: HBL is influenced by both patient-related and surgery-related factors. Greater HBL negatively affects early recovery by prolonging hospitalization, impeding functional recovery, and increasing complication risks. The findings provide a preliminary basis for integrating HBL monitoring into Enhanced Recovery After Surgery (ERAS) pathways. Proactive perioperative blood management is recommended for high-risk patients to improve prognosis.

## 1. Introduction

With the continuous advancement of tumor research and therapeutic methods, the survival period of patients with malignant tumors has been extended, leading to a gradual increase in the incidence of spinal metastases [1,2]. In clinical practice, the primary treatment objectives for spinal metastatic tumors include pain relief, restoration or preservation of neurological function, restoration of spinal stability, and improvement in patient quality of life [3,4]. Surgical intervention, as a key component of comprehensive treatment strategies, plays an irreplaceable role in achieving these objectives [5]. Surgery can directly remove tumor tissue, relieve compression on the spinal cord and nerve roots, and restore spinal stability, thereby effectively alleviating pain and improving neurological deficits [4,6].

However, surgery for spinal metastatic tumors still faces numerous challenges, among which hidden blood loss (HBL), as a frequently overlooked clinical issue, deserves particular attention [7,8]. HBL mainly refers to blood loss during and after surgery through pathways such as wound exudation, retention in tissue spaces, accumulation in body cavities, or hemolysis. Due to its concealed nature, it is often difficult to fully assess [9,10]. In spinal metastasis surgery, due to the extensive surgical scope, significant trauma, and the rich blood supply in the spinal region, blood loss is particularly pronounced [11,12]. Nevertheless, current research on HBL in such surgeries remains relatively limited, and its influencing factors are complex and varied. More importantly, the association between HBL and the patient’s postoperative recovery process has not been systematically elucidated, an issue that directly relates to surgical outcomes and patient prognosis [13]. Therefore, this study retrospectively analyzed patients who underwent surgery for spinal metastatic tumors at our institution, aiming to investigate the volume of HBL and its related influencing factors, and to further evaluate the impact of HBL on patients’ early postoperative recovery status.

## 2. Materials and Methods

### 2.1. Patient Population

This study is a single-center, retrospective cohort study. Clinical medical records of patients who presented with spinal metastases at the Department of Spinal Surgery, Beijing Shijitan Hospital, Capital Medical University, between January 2019 and October 2024, were analyzed. Patients who strictly met the inclusion/exclusion criteria and had complete perioperative data were included in this study. Finally, 230 patients aged 18 years or older, pathologically confirmed with spinal metastatic tumors, were enrolled. Patient data were obtained through the hospital’s medical record management system. Collected information included age, sex, body mass index (BMI), hypertension (blood pressure ≥ 140/90 mmHg), diabetes (fasting blood glucose ≥ 6.1 mmol/L), primary tumor type, number of extraspinal metastases, site of spinal metastasis, vertebral involvement area, use of orthopedic robot-assisted surgery, surgical duration, intraoperative blood loss, preoperative albumin level, preoperative and postoperative hemoglobin levels, and preoperative and postoperative hematocrit. Simultaneously, to assess the impact of HBL on postoperative status, we collected the following indicators: postoperative hospital stay (from surgery day to discharge day), Karnofsky Performance Status (KPS) scores on the day before surgery and on discharge day (which typically occurred within 7–10 days postoperatively unless delayed by complications), and calculated the change value (ΔKPS = postoperative KPS—preoperative KPS). The occurrence of complications during the postoperative hospital stay was recorded, and their severity was classified according to the Clavien–Dindo classification system. All surgeries were performed by the same team of spine surgeons with over 20 years of experience. Informed consent was obtained from all patients for the clinical data involved in this study. All methods were performed in accordance with the relevant guidelines and regulations of Declaration of Helsinki. This study was approved by the Medical Ethics Committee of Beijing Shijitan Hospital affiliated with Capital Medical University (Ethics Approval Number: IIT2024-127-002).

### 2.2. Inclusion and Exclusion Criteria

#### 2.2.1. Inclusion Criteria

① Cases confirmed as spinal metastatic tumors via imaging (e.g., X-ray, CT, MRI) and histopathological examination, with only single-segment spinal involvement (extraspinal metastases were allowed). ② Patients undergoing their first posterior tumor resection, bone cement augmentation, and pedicle screw internal fixation for spinal metastases. ③ Availability of complete preoperative and postoperative blood test records (including at least hemoglobin and hematocrit) and detailed anesthesia records (documenting intraoperative blood loss, transfusion volume, urine output, etc.).

#### 2.2.2. Exclusion Criteria

① Coexisting severe hematological diseases, including but not limited to known coagulation disorders (e.g., hemophilia), platelet count < 100 × 10^9^/L, or diseases severely affecting coagulation function (e.g., liver cirrhosis Child–Pugh class B/C). ② Severe insufficiency of major organ functions: cardiac function NYHA class III–IV; liver function (ALT/AST > 2 times the upper limit of normal); renal function (creatinine clearance rate < 30 mL/min). ③ Use of therapeutic doses of anticoagulants (e.g., warfarin, rivaroxaban) or antiplatelet agents (e.g., aspirin, clopidogrel) within one week before surgery. ④ Receipt of therapeutic doses of low-molecular-weight heparin injections for conditions such as deep vein thrombosis prior to surgery. ⑤ History of previous spinal surgery at the same site. ⑥ Receipt of radiotherapy or embolization therapy targeting the spinal metastasis within one month before surgery.

### 2.3. Surgical Technique

Preoperative planning was based on the Tomita classification for spinal tumors, aiming to achieve an individualized en bloc resection strategy with the goal of obtaining a wide or at least marginal resection margin. All surgeries employed a posterior midline approach, with the main steps including pedicle screw insertion, anterior column augmentation, and tumor resection. Depending on the method of screw insertion, the procedures were divided into the following two scenarios:

#### 2.3.1. Robot-Assisted Pedicle Screw Insertion

After satisfactory general anesthesia, the patient was placed in the prone position. A midline incision was made on the back, sequentially incising the skin, subcutaneous tissue, and supraspinous ligament. Subperiosteal dissection was performed along both sides of the spinous processes to adequately expose the laminae, facet joints, and transverse processes. A locator was installed on the spinous process, and intraoperative three-dimensional CT scanning was performed. The imaging data was imported into the Tianji Orthopedic Robotic System (Beijing Tinavi Medical Technologies Co., Ltd., Beijing, China) for virtual screw trajectory planning. Guided by robotic navigation, a Kirschner wire was inserted as a reference, followed sequentially by pilot hole creation, tapping, and insertion of the pedicle screws.

#### 2.3.2. Conventional Manual Pedicle Screw Insertion

The patient positioning, incision, and exposure steps were the same as described above. After exposing the anatomical structures, manual localization and orientation were performed based on intraoperative fluoroscopic images and anatomical landmarks (such as the transverse processes, facet joints, and laminae). A starter awl was used to manually create the pilot hole. After confirming the integrity of the tunnel walls (four walls and floor) using a probe, the channel was prepared with a tap. The pedicle screws were then inserted, and their position and depth were verified as satisfactory through repeated fluoroscopy.

#### 2.3.3. Subsequent Surgical Procedures

**Figure 1:**
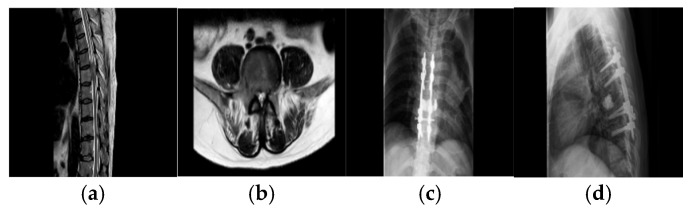
(**a**,**b**): Preoperative MRI demonstrates tumor involvement of the T7 vertebral body. (**c**,**d**): Postoperative anteroposterior and lateral radiographs at 1 week show satisfactory recovery.

Following screw insertion, working cannulas were inserted through openings made at the surface projections of the bilateral pedicles for anterior column augmentation: a balloon was inserted and inflated to expand the vertebral body, followed by slow injection of bone cement under fluoroscopic monitoring. Subsequently, an ultrasonic bone scalpel was used to perform en bloc resection of the tumor tissue along the pre-defined margins, with careful attention to protecting adjacent neurovascular structures. After resection was complete, the surgical field was thoroughly irrigated, a drainage tube was placed, and the incision was closed in layers. Figure 1 illustrates the preoperative imaging evaluation and postoperative internal fixation status in a representative patient.

### 2.4. Management of Blood Loss

The time frame for perioperative blood loss assessment was set from the day before surgery to the day of postoperative drainage tube removal. To accurately calculate HBL, serial complete blood count tests were performed on patients at the following time points: the day before surgery, postoperative days 1, 2, and 3, and subsequently every two days until discharge. The lowest postoperative hemoglobin (Hb) and hematocrit (HCT) values measured during this period, combined with the preoperative baseline values, were used to calculate postoperative anemia and HBL. The definition of postoperative moderate-to-severe anemia followed the World Health Organization standard [14], i.e., Hb < 110 g/L. Additionally, blood drained via the postoperative drainage tube was included in measurable blood loss. Therefore, the total measurable blood loss in this study consisted of the intraoperative blood loss recorded in the anesthesia records and the total volume of postoperative drainage fluid.

All patients adhered to our institution’s established clinical pathway for perioperative blood management. Each patient received an intravenous infusion of tranexamic acid at a single dose of 15 mg/kg prior to skin incision. During surgery, an additional appropriate dose could be administered at the surgeon’s discretion if significant blood loss was anticipated. Postoperative continuous use of tranexamic acid was not part of the routine protocol.

Regarding red blood cell transfusion, the practical indications adopted by our center during such surgeries were as follows: transfusion of packed red blood cells was considered when the patient’s hemoglobin level fell below 80 g/L [15] or when it was above this threshold but the patient exhibited clinical symptoms related to anemia (such as significant tachycardia, hypotension, dizziness, or decreased oxygen saturation) that were unresponsive to fluid resuscitation. The final decision for transfusion was made jointly by the anesthesiologist and the surgical team based on real-time assessment.

### 2.5. Calculation of HBL

First, the patient’s blood volume was calculated using the Nadler formula: Male: PBV (L) = 0.3669 × height (m)^3^ + 0.03219 × weight (kg) + 0.6041; Female: PBV (L) = 0.3561 × height (m)^3^ + 0.03308 × weight (kg) + 0.1833.

Next, the Gross equation was applied to calculate the “theoretical total blood loss” (TBL): TBL (mL) = PBV (L) × (HCT_pre_ − HCT_post_)/HCT_ave_; where HCT_pre_ represents the hematocrit on the day before surgery, HCT_post_ is the lowest postoperative hematocrit, and HCT_ave_ is the average of HCT_pre_ and HCT_post_.

If the patient received a blood transfusion during this period, the volume of transfused red blood cells was added back: TBL_adj_ (mL) = TBL (mL) + BTV (mL). Finally, HBL was calculated by subtracting the measurable blood loss (intraoperative + postoperative): HBL (mL) = TBL_adj_ (mL) − MBL (mL). Intraoperative blood loss was obtained from the anesthesiologist’s intraoperative fluid management records.

### 2.6. Vertebral Metastasis

**Figure 2:**
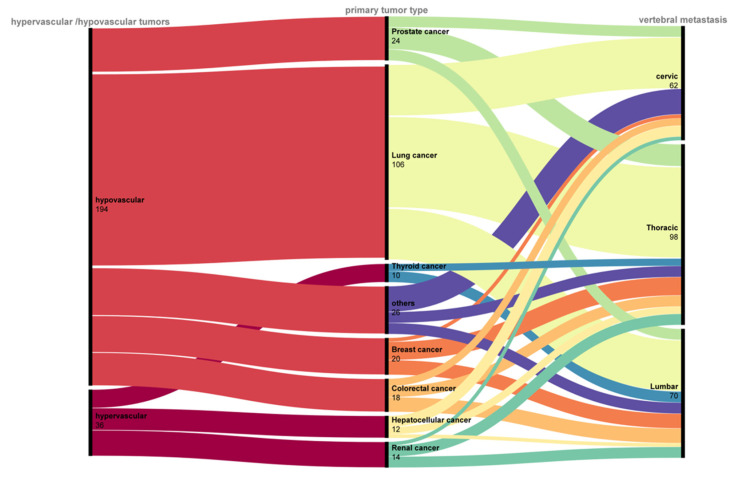
Illustration of primary tumor types, origin, and metastatic sites.

The cohort in this study comprised patients with isolated, single-segment metastases in the cervical, thoracic, or lumbar spine. Figure 2 illustrates the distribution of these metastatic lesions across vertebral levels, as well as the proportion of hypervascular and non-hypervascular primary tumor types.

**Figure 3:**
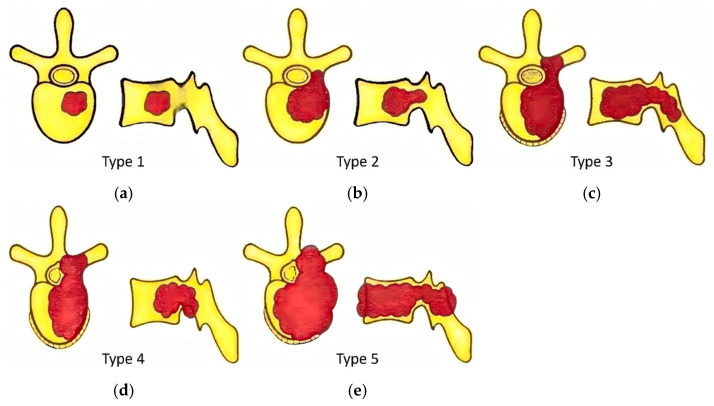
(**a**) Type 1—localized inside the body or lamina; (**b**) Type 2—the lesion extends into the pedicle; (**c**) Type 3—the extension is throughout the vertebra; (**d**) Type 4—there is epidural extension; (**e**) Type 5—the paraspinal area is affected. As illustrated, the red areas indicate the tumor-involved regions.

All included cases underwent preoperative vertebral CT and MRI examinations and were classified as Tomita type 1–5 for spinal metastatic tumors based on the imaging findings [16], as shown in Figure 3.

### 2.7. Statistical Analysis

Statistical analysis was performed using SPSS Statistics software (version 27.0; IBM Corp., Armonk, NY, USA) and GraphPad Prism (version 10.6.0; GraphPad Software, San Diego, CA, USA). Continuous variables were first assessed for distribution using the Shapiro–Wilk test combined with histograms/Q–Q plots. Normally distributed data are presented as mean ± standard deviation, while non-normally distributed data are presented as median (Q1, Q3). The primary outcome was the influencing factors of postoperative HBL (HBL, mL). Univariate regression was employed, and a multivariate model included clinically predefined covariates (age, sex, BMI, preoperative Hb, Tomita classification, surgical duration, metastasis site, etc.), with collinearity assessed (VIF). Secondary outcomes involved evaluating the association between HBL and postoperative outcomes: (1) length of hospital stay and ΔKPS as continuous outcomes, analyzed using multivariate linear regression; (3) anemia severity as a binary outcome, analyzed using logistic regression; (4) Clavien–Dindo complication grade as an ordinal outcome, analyzed using ordinal logistic regression with testing of the proportional odds assumption. Furthermore, to explore potential clinical decision thresholds for HBL and integrate its quantitative assessment into the postoperative management pathway, we conducted Receiver Operating Characteristic (ROC) curve analysis. Based on the core clinical outcomes of interest in this study, we selected postoperative complications (Clavien–Dindo grade ≥ II) and postoperative moderate-to-severe anemia (Hb < 110 g/L) as state variables, with HBL as the test variable. The optimal HBL cut-off value (i.e., the value at which the Youden index is maximized) for predicting the aforementioned adverse events was determined via the ROC curve, and the corresponding area under the curve (AUC) was calculated. All effect sizes are reported with 95% CI, and *p* < 0.05 was considered statistically significant.

## 3. Results

A total of 230 patients were retrospectively studied. Among them, 146 were male, and 84 were female, with a mean age of 63.89 ± 12.56 years (range 18–86 years). Table 1 presents the patients’ demographic and clinical characteristics. The mean body mass index (BMI) was 23.62 ± 3.76 kg/m^2^, and the mean patient blood volume (PBV) was 4.24 ± 0.73 L. Intraoperative allogeneic red blood cell transfusion was administered to 116 patients (50.4%). The preoperative mean hemoglobin (Hb) and hematocrit (HCT) were 124.66 ± 15.86 g/L and 0.37 ± 0.05, respectively. The postoperative mean lowest Hb and HCT were 110.62 ± 16.12 g/L and 0.33 ± 0.05, respectively. The mean HBL was 449.87 ± 284.86 mL, the mean measurable blood loss (MBL) was 621.90 ± 259.13 mL, and the mean total blood loss (TBL) was 1212.93 ± 493.33 mL. Comparisons of preoperative and postoperative HCT (*p* < 0.001) and Hb (*p* < 0.001) showed statistically significant differences (Table 2).

### 3.1. Primary Outcome (Analysis of Influencing Factors for HBL)

Univariate linear regression analysis of the above influencing factors revealed that BMI (*p* < 0.001), surgical duration (*p* < 0.001), robot-assisted surgery (*p* = 0.001), spinal metastasis site, vertebral involvement area, preoperative comorbidities, and preoperative hemoglobin level (*p* = 0.017) were correlated with HBL, as shown in Table 3.

Subsequently, multivariate linear regression analysis of the above factors related to HBL was performed. The results showed that BMI (*p* = 0.003), surgical duration (*p* = 0.026), vertebral involvement area (*p* < 0.001), and preoperative comorbidities (diabetes and/or hypertension) (*p* = 0.032) were independent influencing factors for HBL. Other factors did not show a significant correlation with HBL, as presented in Table 4.

### 3.2. Secondary Outcomes (Impact of HBL on Early Postoperative Outcomes)

To evaluate the clinical impact of HBL, we further analyzed the relationship between HBL and four early postoperative outcome indicators. The results of the correlation analysis are summarized in Table 5.

**Figure 4:**
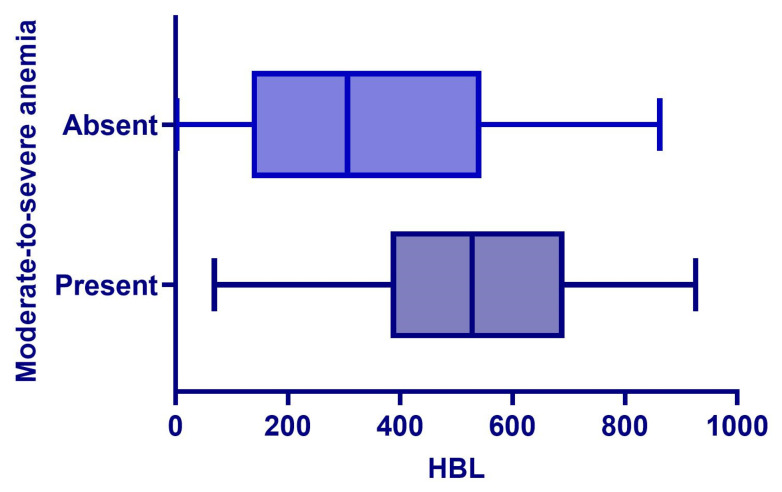
Relationship between HBL and postoperative moderate-to-severe anemia.

**Figure 5:**
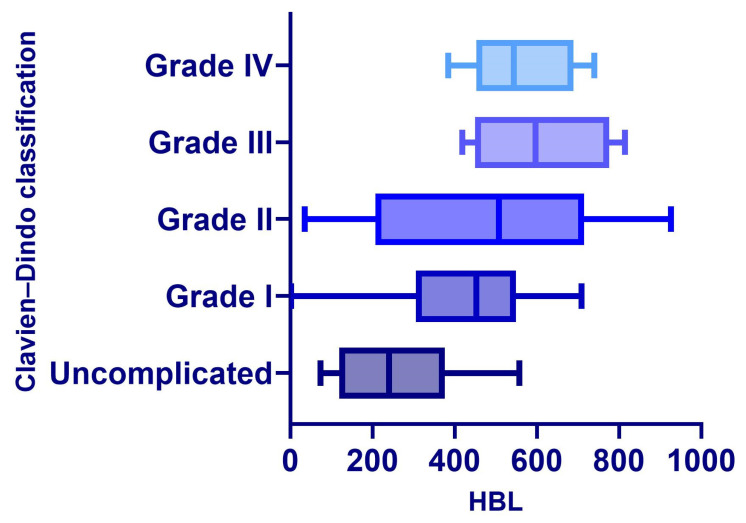
Relationship between HBL and postoperative complication grades.

After adjusting for confounding factors such as age, preoperative KPS score, Tomita classification, surgical duration, and intraoperative MBL, multivariate linear regression analysis showed that HBL was an independent positive predictor of postoperative length of hospital stay (β = 0.023, 95% CI: 0.016–0.030, *p* < 0.001). Concurrently, HBL was significantly negatively correlated with the change in postoperative functional status score (ΔKPS) (β = −0.012, 95% CI: −0.016~−0.008, *p* < 0.001). Furthermore, HBL was also an independent risk factor for the occurrence of postoperative moderate-to-severe anemia (OR = 1.002, 95% CI: 1.001~1.003, *p* < 0.001). In multivariate logistic regression analysis, a higher HBL level was significantly associated with an increased risk of overall postoperative complications (OR = 1.003, 95% CI: 1.001~1.005, *p* < 0.001), as shown in Table 6. Figure 4 and Figure 5 respectively demonstrate the relationship between HBL and postoperative moderate-to-severe anemia, as well as the relationship between HBL and complication grades.

**Figure 6:**
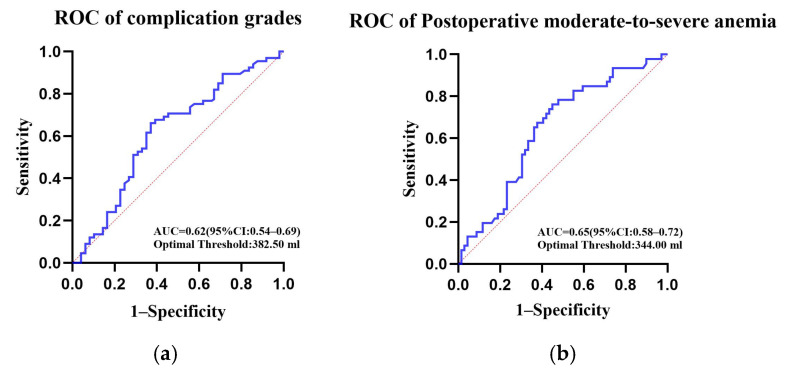
(**a**) HBL predicting complications (Grade ≥ II); (**b**) HBL predicting moderate-to-severe anemia.

ROC curve analysis demonstrated that HBL exhibited a certain predictive ability for postoperative complications (≥Clavien–Dindo Grade II), with an AUC of 0.62 (95% CI: 0.54–0.69, *p* = 0.002). The optimal threshold was 382.50 mL based on the Youden index, as shown in Figure 6a. Simultaneously, HBL also showed good predictive capability for postoperative moderate-to-severe anemia, with an AUC of 0.65 (95% CI: 0.58–0.72, *p* = 0.002). Its optimal threshold was 344.00 mL, as shown in Figure 6b.

## 4. Discussion

Current research on HBL after spinal surgery has primarily focused on procedures such as scoliosis correction, vertebroplasty, kyphoplasty, and interbody fusion [8,17,18,19], while studies specifically investigating HBL following surgery for spinal metastases are relatively scarce. This may be because many studies have prioritized the investigation of immediately life-threatening factors, such as intraoperative measurable bleeding. For instance, Mohme et al., in a large retrospective study, reported an average intraoperative blood loss of 1176 ± 1209 mL in patients with spinal metastases, and found that tumor histology, surgical duration, Epidural Spinal Cord Compression (ESCC) score, BMI, and surgical approach were significantly associated with an increased risk of intraoperative blood loss [20]. An earlier meta-analysis indicated an average intraoperative blood loss of 2180 mL for spinal metastasis surgery [21]. In contrast, the role of HBL, as an important postoperative complication, has not received sufficient attention in the context of surgery for spinal metastases and the subsequent postoperative period.

In fact, as a common complication following various spinal surgeries, the clinical importance of HBL has been widely recognized. A retrospective study by Raitio et al. showed that among 57 pediatric patients with congenital scoliosis undergoing spinal osteotomy, HBL accounted for an average of 40% of TBL [19]. Additionally, a study on transforaminal lumbar interbody fusion indicated that the average HBL could account for 42% of TBL [22]. Therefore, to further clarify its significance in spinal metastasis surgery, this study investigated from two perspectives. The results demonstrated that postoperative HBL accounted for approximately 37% of TBL in spinal metastasis surgery, which aligns with previous reports, further confirming the prevalence of this phenomenon. Moreover, this study identified BMI, surgical duration, vertebral involvement area, and hypertension as independent influencing factors for HBL. More importantly, this study elucidated that HBL is not only a significant component of total postoperative blood loss but also an independent risk factor affecting patients’ early recovery outcomes. This is specifically manifested as prolonged postoperative hospital stay, delayed recovery of functional status, increased risk of moderate-to-severe anemia, and increased overall severity of complications.

It should be noted, however, that current evidence on HBL after spinal metastasis surgery, like much of the aforementioned similar research, primarily originates from small-sample, single-center observational studies, with a relative scarcity of high-quality randomized controlled trials. This to some extent limits the robustness and generalizability of the relevant conclusions. Against this background, this study, as a single-center retrospective analysis, aims to contribute descriptive data to this field through a relatively large cohort and to explore potential associations.

In our study, surgical duration was identified as an independent influencing factor for HBL, a finding consistent with numerous reports across various surgical fields. For example, Yang et al., in a study on percutaneous vertebroplasty for osteoporotic vertebral compression fractures, noted that longer surgical duration significantly increases HBL [23]; Guo et al.’s report on unilateral biportal endoscopic surgery for lumbar spinal stenosis also suggested similar conclusions [24]. The underlying mechanism may be that prolonged surgical duration corresponds to an increased exposure time of the wound to the external environment, thereby extending the duration of tissue fluid exudation and chronic oozing, ultimately leading to more significant HBL.

Our analysis suggests that the formation of HBL may involve a combination of surgery-related factors and patient-specific factors. Among the latter, BMI, as an important quantifiable indicator, was confirmed to be an independent influencing factor for HBL. Furthermore, a study by Miao et al. also demonstrated a positive correlation between HBL and changes in BMI among 322 patients undergoing total hip arthroplasty [25]. However, some previous studies have suggested that BMI is not an influencing factor for HBL [17,26]. This inconsistency may stem from multiple reasons. Firstly, the heterogeneity of study populations is a key factor. Compared to patients with degenerative diseases, those with spinal metastases in this study have more complex surgical extents, tumor vascularity, and overall systemic conditions, potentially amplifying the effects of high BMI, such as difficulties in surgical exposure and increased tissue bleeding. Secondly, BMI itself is a crude indicator that cannot differentiate between fat and muscle proportions, whose impacts on bleeding may differ [27]. Future prospective, large-sample studies, combined with precise measurements such as body composition analysis, are still needed to further clarify the exact role of BMI in perioperative blood loss.

Among the various influencing factors, particular attention should be paid to the vertebral involvement area, which, as a unique indicator for spinal metastases, has been confirmed in this study to be an important factor in predicting HBL. Specifically, patients with vertebral involvement classified as Tomita type 4 or type 5 exhibited higher HBL, consistent with the prediction by Li et al. that a larger surgical extent is closely associated with greater blood loss in patients with spinal metastases [12]. In our study, “type 4 or type 5” metastases indicate more extensive infiltration of the tumor within the vertebral body and surrounding structures, meaning that, in addition to involving the entire vertebra, there is epidural extension or paraspinal area involvement. Consequently, to achieve radical tumor resection, the required surgical extent inevitably expands significantly, directly resulting in more extensive bone and soft tissue wounds, which provide a larger surface area for blood exudation. Secondly, the growth of malignant tumors relies on an abnormally abundant and fragile pathological vascular network. The higher tumor burden represented by “type 4 or type 5” is often accompanied by more developed pathological blood supply. Studies have indicated that intraoperative blood loss is significantly higher in hypervascular spinal metastases, such as those from renal or thyroid cancers [28].

While focusing on local anatomical factors, the patient’s systemic baseline condition is also a significant factor influencing perioperative blood loss. This study found that preoperative hypertension is an independent risk factor for HBL, whereas diabetes did not show a significant association with HBL. This suggests that, in addition to surgery-related local factors, the patient’s physiological reserve capacity and vascular functional status play an important role in the mechanism of HBL. This finding is consistent with some existing research results [18,29]. However, other studies have suggested that both diabetes and hypertension are influencing factors for postoperative HBL [17], and some reports even indicate that neither has a significant impact on HBL [8]. We speculate that the reasons for these inconsistent conclusions may include the following aspects: First, the types of surgeries included in different studies vary, such as vertebroplasty, kyphoplasty, lumbar interbody fusion, and intertrochanteric fracture surgery, each with differences in the extent of surgical trauma and bleeding characteristics. Second, the patient populations across studies exhibit significant heterogeneity in terms of the severity, duration, treatment standardization, and comorbidities of underlying diseases. For example, factors such as blood pressure control levels in hypertensive patients, the quality of glycemic management in diabetic patients, and the presence of microvascular complications may all interfere with the assessment of HBL. Furthermore, the definitions, measurement methods, and calculation formulas for HBL are not uniform across studies, and differences in perioperative management strategies—such as fluid resuscitation protocols, transfusion thresholds, and anticoagulant use—may also lead to biases in blood loss assessment. Therefore, when interpreting existing evidence, the impact of clinical and methodological heterogeneity on research conclusions should be fully considered. Future efforts should promote more rigorously designed, large-sample studies targeting specific surgical procedures to systematically clarify the specific mechanisms by which systemic factors such as hypertension and diabetes contribute to perioperative HBL.

It is noteworthy that, through multivariate analysis, our study did not identify primary tumor type as an independent influencing factor for postoperative HBL. This result differs from the conclusions of some literature focusing on intraoperative measurable blood loss, which often suggests that hypervascular metastases such as renal cell carcinoma and thyroid carcinoma are associated with a higher risk of intraoperative bleeding [28,30]. This discrepancy may stem from the fundamental differences in the mechanisms and time windows between HBL and intraoperative measurable blood loss: HBL primarily manifests as persistent postoperative oozing into tissue spaces, and its volume is more likely influenced by the extent of surgical trauma, surgical duration, and the patient’s systemic condition (e.g., BMI, hypertension), rather than the vascular richness of the tumor itself. Furthermore, for hypervascular metastases, routine clinical measures such as preoperative prophylactic embolization and targeted intraoperative hemostasis may have effectively controlled the active bleeding primarily originating from the tumor-feeding arteries, thereby reducing their impact during the postoperative HBL phase, which is dominated by chronic exudation [31].

Our study confirms that postoperative HBL is an independent risk factor for the development of moderate-to-severe anemia (Hb < 110 g/L) in patients. This conclusion aligns with previous studies, all indicating that concealed blood volume loss can directly lead to decreases in hemoglobin and hematocrit [32,33]. Tonino et al. further noted that changes in hemoglobin levels affect postoperative recovery outcomes [34], and this effect is more pronounced when hemoglobin levels are lower. Additionally, studies have shown that anemia can reduce effective circulating blood volume and total red blood cell mass, thereby impairing blood oxygen-carrying capacity and resulting in inadequate tissue oxygen supply [35]. These mechanisms not only provide a pathophysiological explanation for symptoms such as fatigue and reduced exercise tolerance observed in patients after surgery but also lay the foundation for the development of other adverse recovery outcomes.

Further empirical analysis demonstrates that HBL directly delays the process of postoperative physiological and functional recovery in patients by inducing or exacerbating anemia. In previous prognostic assessments, both performance status and length of hospital stay have been regarded as critical factors affecting patient survival [36,37]. This study, through multivariate linear regression, confirmed that HBL is a positive predictor of postoperative hospital stay and shows a significantly negative correlation with ΔKPS. This indicates that greater HBL is associated with longer hospital stays and slower functional recovery in patients. Based on this, we hypothesize that the underlying mechanism may be as follows: anemia induced by increased HBL leads to reduced physical capacity and significantly diminished rehabilitation motivation and participation in patients. Slow recovery of functional status directly affects discharge assessments, prolonging observation and intervention cycles, thereby creating a clinical positive feedback loop of “HBL → anemia → delayed functional recovery → prolonged hospital stay.” However, it is important to contextualize the finding regarding prolonged hospital stay. The absolute length of stay is heavily influenced by institution-specific discharge criteria, clinical pathways, and resource availability, as is the case in our and most other single-center studies in this field. Therefore, while the association between HBL and delayed discharge is mechanistically sound, the generalizability of the magnitude of this effect to healthcare settings with different discharge protocols or economic models may be limited.

Simultaneously, the results of this analysis indicate that a higher volume of HBL is significantly associated with an increased risk of overall postoperative complications. Perioperative anemia has been confirmed to be closely related to various complications, including rebleeding, impaired wound healing, and compromised motor function [38]. The underlying mechanisms may be multifaceted: first, inadequate tissue oxygen supply caused by anemia can weaken tissue repair capacity and impair immune function, thereby increasing the risk of surgical site infections and poor wound healing [39]; second, to compensate for the reduced oxygen-carrying capacity due to anemia, the load on the cardiovascular system is continuously heightened, which may trigger cardio-cerebrovascular events such as myocardial ischemia and arrhythmias [40].

Currently, there is no established consensus on ERAS protocols for complex spinal procedures such as surgery for spinal metastases [41]. Against this backdrop, the present retrospective analysis observed a possible association between hidden blood loss (HBL) and adverse postoperative outcomes. ROC analysis indicated that an HBL > 382.5 mL was correlated with a trend toward an increased risk of complications requiring intervention, while an HBL > 344.0 mL appeared to show a relatively stronger association with the occurrence of moderate-to-severe postoperative anemia.

Based on these observational findings, we tentatively propose a preliminary conceptual framework for integrating HBL monitoring into perioperative management. This framework should be regarded as an exploratory scheme intended to generate hypotheses. Within this concept, we introduce a clinically convenient reference alert value: 360 mL. This rounded figure is derived from the two aforementioned ROC-derived thresholds (382.5 mL and 344.0 mL), and its potential utility lies in suggesting the consideration of more proactive perioperative blood management assessments and intensified postoperative monitoring:

1. Preoperative Phase: Focus on identifying patients with high-risk factors, such as high BMI, Tomita type 4–5 lesions, and preoperative hypertension, to conduct risk assessment and formulate individualized management plans.
2. Intraoperative Phase: Emphasize meticulous surgical technique and definitive hemostasis, particularly for cases with extensive vertebral involvement (Tomita type 4–5), to reduce both visible and hidden blood loss at the source.
3. Postoperative Phase: Implement HBL-guided dynamic management. For high-risk patients with HBL > 360 mL, management should be escalated to include: ① Enhanced Monitoring: Increasing the frequency of hemoglobin checks (e.g., daily until stabilization) to detect covert anemia early. ② Preemptive Intervention: Early initiation of intravenous iron or erythropoietin therapy to proactively correct anemia and prevent associated fatigue and delayed functional recovery. ③ Individualized Rehabilitation: Adjusting early mobilization and rehabilitation plans based on changes in KPS scores, and carefully determining the optimal timing for discharge.

Through this multi-phase, structured integration concept, we aim to provide a theoretical construct for potentially interrupting the vicious cycle of “hidden blood loss → anemia → delayed functional recovery → complications.” It must be strongly emphasized that this framework originates from single-center retrospective data. Consequently, the suggested associations, the generalizability of the proposed 360 mL threshold, the feasibility of this management framework, and its actual impact on improving patient outcomes all require further verification and evaluation in prospective studies or well-designed quality improvement projects.

Our study has the following limitations. First, its single-center retrospective design carries inherent risks of selection bias and unmeasured confounding factors, which limits causal inference and may restrict the generalizability of the conclusions. Second, certain key perioperative variables were not standardized or comprehensively documented, such as specific hemostatic techniques and history of preoperative radiotherapy or targeted therapy. Furthermore, standardized data on specific complications such as venous thromboembolism were not systematically collected, which limits the exploration of the association between HBL and these important outcomes. These omissions may represent sources of residual confounding. Finally, the proposed framework for integrating HBL into the ERAS pathway remains an exploratory concept, and currently, a lack of consensus exists regarding ERAS protocols for such complex spinal surgeries. Therefore, the feasibility, effectiveness, and generalizability of our HBL threshold-based recommendations require validation in prospective studies. In summary, this study, along with much of the existing evidence in this field, is primarily derived from observational data, which necessitates cautious interpretation of the correlative findings.

## 5. Conclusions

This single-center retrospective analysis suggests that higher body mass index, longer surgical duration, extensive vertebral involvement (Tomita types 4–5), and preoperative hypertension are factors associated with increased hidden blood loss (HBL) following surgery for spinal metastases. More importantly, HBL itself was independently associated with early adverse outcomes, including prolonged hospital stay, attenuated improvement in functional status (KPS), and increased risks of moderate-to-severe anemia and complications. These findings lend support to the clinical relevance of monitoring HBL. However, it is crucial to emphasize that the current evidence on HBL in spinal metastasis surgery, to which this study contributes, is predominantly derived from observational research. Consequently, our conclusions should be viewed as hypothesis-generating and reflect the strength of this preliminary evidence base. Based on these observations, we propose that, for identified high-risk patients, clinical practice could consider implementing a more proactive perioperative blood management strategy, encompassing early monitoring and intervention for HBL and anemia. The ultimate efficacy of this strategy and its feasibility for integration into Enhanced Recovery After Surgery pathways require further validation in prospective studies.

## Figures and Tables

**Table 1:** Patient demographics.

Parameters	Statistics
Gender (*n*,%) Gender	
Male	146 (63.4)
Female	84 (36.6)
Primary tumor type (*n*,%)	
Lung cancer	106 (46.1)
Prostate cancer	24 (10.4)
Breast cancer	20 (8.7)
Colorectal cancer	18 (7.8)
Renal cancer	14 (6.1)
Hepatocellular cancer	12 (5.2)
Thyroid cancer	10 (4.3)
Others	26 (11.4)
Comorbidity (*n*,%)	
Neither diabetes nor hypertension	126 (54.7)
Only Hypertension	54 (23.5)
Only diabetes	22 (9.6)
Both diabetes and hypertension	28 (12.2)
Vertebral involvement (*n*,%)	
Type 1	112 (48.7)
Type 2	16 (7.0)
Type 3	18 (7.8)
Type 4	38 (16.5)
Type 5	46 (20.0)
Intraoperative blood transfusion (*n*,%)	
Yes	116 (50.4)
No	114 (49.6)
Robotic assistance (*n*,%)	
Yes	90 (39.1)
No	140 (60.9)
Vertebral metastasis site (*n*,%)	
Cervical	62 (27.0)
Thoracic	98 (42.6)
Lumbar	70 (30.4)
Number of extraspinal metastases (*n*,%)	
0	113 (49.1)
1–2	75 (32.6)
≥3	42 (18.3)
Postoperative moderate-to-severe anemia (*n*,%)	
Yes	92 (40.0)
No	138 (60.0)
Postoperative complications (*n*,%)	
Uncomplicated	68 (29.6)
Grade I	28 (12.2)
Grade II	106 (46.1)
Grade III	16 (7.0)
Grade IV	12 (5.1)
Postoperative drainage volume (mL)	364.90 ± 147.56
Average age (years)	63.89 ± 12.56
BMI (kg/m^2^)	23.62 ± 3.76
Patient’s blood volume (L)	4.24 ± 0.73
Total blood loss (mL)	1212.93 ± 493.33
Measurable blood loss (mL)	621.90 ± 259.13
HBL (mL)	449.87 ± 284.86
Preoperative hemoglobin level (g/L)	124.66 ± 15.86
Postoperative hemoglobin level (g/L)	110.62 ± 16.12
Preoperative albumin level (g/L)	37.53 ± 4.87
Preoperative hematocrit	0.37 ± 0.05
Postoperative hematocrit	0.33 ± 0.05
Surgical duration (h)	2.76 ± 1.97

**Table 2:** Changes in HCT and HB levels before and after surgery.

Factors	SD	T	*p*
Preoperative and postoperative HB, g/L	13.78	15.44	<0.001
Preoperative and postoperative HCT	0.04	18.75	<0.001

**Table 3:** Results of univariate linear regression for HBL.

Influencing Factors	Unstandardized		Standardized	t	*p*
	β	Standard Error	β		
Gender (*n*,%) Gender					
Female	0	-	0	-	-
Male	47.134	39.069	0.080	1.206	0.229
Age	−1.693	1.506	−0.074	−1.124	0.262
BMI	19.378	4.885	0.254	3.967	<0.001
Surgical duration	79.728	8.026	0.551	9.934	<0.001
Robotic assistance	125.054	37.770	0.214	3.311	0.001
Primary tumor type					
Non-hypervascular	0	-	0	-	-
Hypervascular	89.784	51.596	0.114	1.740	0.083
Vertebral metastasis site					
Cervical	0	-	0	-	-
Thoracic	169.263	44.946	0.294	3.766	<0.001
Lumbar	44.091	48.304	0.071	0.913	0.362
Extraspinal metastases					
0	0	-	0	-	-
1–2	−87.050	42.224	−0.143	−2.061	0.052
≥3	−19.930	51.741	−0.027	−0.385	0.700
Vertebral involvement					
Type 1	0	-	0	-	-
Type 2	68.536	59.304	0.061	1.156	0.249
Type 3	213.327	56.348	0.201	3.786	<0.001
Type 4	372.766	41.658	0.486	8.948	<0.001
Type 5	408.009	38.859	0.573	10.500	<0.001
Comorbidity					
Neither	0	-	0	-	-
Only hypertension	100.582	46.093	0.150	2.182	0.030
Only diabetes	−34.846	65.481	−0.036	−0.532	0.595
Both	−26.508	59.207	−0.030	−0.448	0.655
Preoperative hemoglobin level	2.810	1.173	0.157	2.396	0.017
Preoperative albumin level	3.589	3.895	0.061	0.921	0.358

**Table 4:** Results of multivariate linear regression for HBL.

Influencing Factors	Unstandardized		Standardized	t	*p*
	β	Standard Error	β		
BMI	12.434	3.994	0.164	3.113	0.002
Surgical duration	21.634	12.370	0.150	1.749	0.042
Robotic assistance	24.598	31.090	0.042	0.791	0.430
Vertebral metastasis site					
Cervical	0	-	0	-	-
Thoracic	7.051	40.561	0.012	0.174	0.862
Lumbar	49.665	39.265	0.081	1.256	0.207
Vertebral involvement					
Type 1	0	-	0	-	-
Type 2	48.216	63.116	0.043	0.764	0.446
Type 3	164.339	65.267	0.156	2.518	0.013
Type 4	294.630	54.394	0.378	5.417	<0.001
Type 5	319.982	62.354	0.452	5.132	<0.001
Comorbidity					
Neither	0	-	0	-	-
Only Hypertension	83.437	35.851	0.125	2.327	0.021
Only diabetes	12.039	52.644	0.012	0.229	0.819
Both	21.738	46.035	0.025	0.472	0.637
Preoperative hemoglobin level	0.481	0.929	0.027	0.518	0.605

**Table 5:** Results of the Pearson or Spearman correlation analysis between early postoperative outcomes and HBL.

Early Postoperative Outcomes	*Sig*	*p*
ΔKPS	−0.397	<0.001
Postoperative complications	0.221	<0.001
Postoperative length of hospital stay	0.402	<0.001
Postoperative moderate-to-severe anemia	−0.235	<0.001

**Table 6:** Multivariate analysis of the impact of HBL on postoperative outcomes.

Outcome	Effect Size (95% CI)	*p*
ΔKPS	β = −0.012 (−0.016 ~ −0.008)	<0.001
Postoperative length of hospital stay	β = 0.023 (0.016~0.030)	<0.001
Postoperative moderate-to-severe anemia	OR = 1.002 (1.001~1.003)	<0.001
Postoperative complications		
Grade I	OR = 1.003 (1.002~1.005)	<0.001
Grade II	OR = 1.003 (1.001~1.004)	<0.001
Grade III	OR = 1.003 (1.001~1.005)	<0.001
Grade IV	OR = 1.002 (1.000~1.004)	0.100

## Data Availability

Due to privacy restrictions, the data are not publicly available. However, they can be obtained from the corresponding author (X.F.) upon reasonable request.

## Abbreviations

HBL	Hidden Blood Loss
BMI	Body Mass Index
KPS	Karnofsky Performance Status
PBV	Patient’s Blood Volume
TBL	Total Blood Loss
MBL	Measurable Blood Loss
Hb	Hemoglobin
HCT	Hematocrit
ESCC	Epidural Spinal Cord Compression
ERAS	Enhanced Recovery After Surgery
